# Wisconsin Society of Veterinary Graduates

**Published:** 1900-04

**Authors:** W. G. Clark

**Affiliations:** Secretary


### WISCONSIN SOCIETY OF VETERINARY GRADUATES.

THE annual meeting of the Society was held in Madison, March 14th, at the Hotel Van Etta. The meeting was called to order by the President, Dr. D. Roberts, at 2 o'clock P.M. Present: Drs. B. L. Clark, W. G. Clark, A. H. Hartwig, R. S. Heer, S. S. Snyder, G. Ed. Leech, D. Roberts, E. D. Roberts, and Charles Schmitt. Visitors: Drs. Simon Beattie, Madison, and Walter Pick, Lodi. The minutes of the last meeting were read and approved.

The Secretary's report of accounts was read and accepted. The Treasurer's report was read and accepted.

Dr. Hartwig brought up the subject of prosecutions under the new law; discussed by Drs. Leech, Heer, and Schmitt.

Under the head of new business, a communication from Dr. W. E. A. Wyman tendering his resignation from the Society was read and discussed by Drs. Leech, Schmitt, Clark, Snyder, and Hartwig. On motion Dr. Wyman's resignation was accepted. On motion, the Secretary was instructed to retain Dr. Wyman's certificate of membership and return his membership fee. It was moved by Dr. Leech and seconded by Dr. Hartwig, that the President and Secretary be empowered as a committee to restrict Dr. Wyman from using the Society or the Society's interests in any way to further his personal interests, and that the President and Secretary be given the power of attorney to prosecute the case if necessary; carried.

The application of Drs. S. Beattie and W. R. Pick being reported on favorably by the Censors, they were balloted on and declared elected to membership.

The Secretary was requested to read Section 7 of the Code of Ethics in regard to live-stock insurance companies. The Secretary then read a report of the financial condition of the Badger Mutual Live-stock Insurance Company of Milwaukee. Discussed by Drs. Leech, Hartwig, Clark, and Schmitt. Moved by Dr. Leech and seconded by Dr. Heer, that the Secretary forward to all members of the Society not present a marked copy of the Constitution and By-laws.

On motion the Society proceeded to the election of officers. Moved and seconded, that the election be by *viva voce* vote; carried. The election resulted as follows: President, A. H. Hartwig, Watertown; Vice-president, J. F. Roub, Monroe; Secretary, W. G. Clark, Marinette; Treasurer, S. S. Snyder, Cedarsburg; Censors, H. P. Clute, Marinette; R. S. Heer, Plattville, and B. L. Clark, Monticello.

On motion it was decided to hold the semi-annual meeting at Milwaukee in the month of September.

On motion, Drs. Ormond, Leech, and D. Roberts were appointed a committee to make arrangements for the semi-annual meeting.

On motion, the Society adjourned, to meet at eight o'clock.

*Evening Session.*

Meeting called to order at 8.30 by the President, Dr. A. H. Hartwig.

The first subject on the programme was a paper on "Periodic Ophthalmia," by Dr. G. Ed. Leech, Milwaukee. It was carefully prepared and presented the latest researches in both human and veterinary pathology in regard to this disease. Discussed by Drs. E. D. Roberts, Hartwig, Schmitt, and D. Roberts. On motion, the discussion was closed and a vote of thanks tendered the essayist.

In the absence of Dr. Wyman, his paper on "Mesoperoneal and Double Peroneal Neurectomy" was read by the Secretary. In the absence of Dr. Wyman the paper was not discussed.

Drs. Leech, Heer, and E. D. Roberts discussed "neurectomy."

The use of eserine sulphate, barium chloride, etc., were quite fully discussed. Dr. Leech recommended oil of eucalyptus in drachm doses hypodermatically or orally for intestinal flatulence.

The use of potassium iodide in the treatment of azoturia was discussed. Dr. D. Roberts reported favorable results when combined with sodium bicarbonate. Drs. Leech, Heer, and Hartwig reported unfavorable results from the treatment.

On motion, the Society adjourned to meet at Dr. Beattie's hospital for clinics at 8.30 the next morning.

CLINICS.

Dr. Beattie presented a case that had showed difficulty in respiration, gradually increasing for a year or more. On examination the horse was found to have suffered a fracture of the nasal bone. This case was not operated on, as the weather was very cold.

Dr. D. Roberts performed ovariotomy on a bitch, and did the operation very quickly and neatly.

Dr. H. P. Clute gave a demonstration in veterinary dentistry, after which the Society adjourned to meet in Milwaukee next September.

W. G. Clark,
Secretary.

